# Supplementary material for: Causal Relationship between Aging and Anorexia Nervosa: A White-Matter-Microstructure-Mediated Mendelian Randomization Analysis
Source: Biomedicines. 2024 Aug 16;12(8):1874. doi: 10.3390/biomedicines12081874 (PMC11351342; doi:10.3390/biomedicines12081874)
Supplement: Supplementary file 1 [file biomedicines-12-01874-s001.zip › Supplementary Figure/Figure S4/Singe SNP plot of MR analysis for TL to ubm-b-1621.pdf]

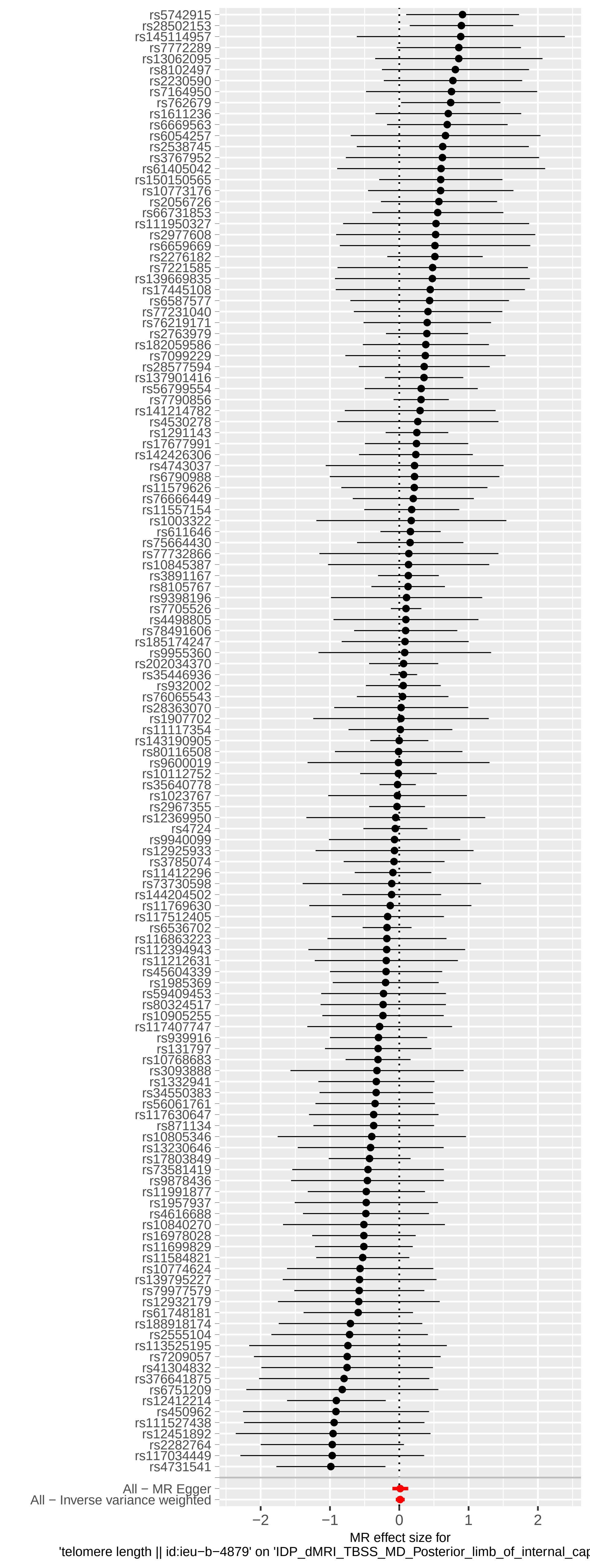
rs5742915
rs28502153
rs145114957
rs7772289
rs13062095
rs8102497
rs2230590
rs7164950
rs762679
rs1611236
rs6669563
rs6054257
rs2538745
rs3767952
rs61405042
rs150150565
rs10773176
rs2056726
rs66731853
rs111950327
rs2977608
rs6659669
rs2276182
rs7221585
rs139669835
rs17445108
rs6587577
rs77231040
rs76219171
rs2763979
rs182059586
rs7099229
rs28577594
rs137901416
rs56799554
rs7790856
rs141214782
rs4530278
rs1291143
rs17677991
rs142426306
rs4743037
rs6790988
rs11579626
rs76666449
rs11557154
rs1003322
rs611646
rs75664430
rs77732866
rs10845387
rs3891167
rs8105767
rs9398196
rs7705526
rs4498805
rs78491606
rs185174247
rs9955360
rs202034370
rs35446936
rs932002
rs76065543
rs28363070
rs1907702
rs11117354
rs143190905
rs80116508
rs9600019
rs10112752
rs35640778
rs1023767
rs2967355
rs12369950
rs4724
rs9940099
rs12925933
rs3785074
rs11412296
rs73730598
rs144204502
rs11769630
rs117512405
rs6536702
rs116863223
rs112394943
rs11212631
rs45604339
rs1985369
rs59409453
rs80324517
rs10905255
rs117407747
rs939916
rs131797
rs10768683
rs3093888
rs1332941
rs34550383
rs56061761
rs117630647
rs871134
rs10805346
rs13230646
rs17803849
rs73581419
rs9878436
rs11991877
rs1957937
rs4616688
rs10840270
rs16978028
rs11699829
rs11584821
rs10774624
rs139795227
rs79977579
rs12932179
rs61748181
rs188918174
rs2555104
rs113525195
rs7209057
rs41304832
rs376641875
rs6751209
rs12412214
rs450962
rs111527438
rs12451892
rs2282764
rs117034449
rs4731541
All − MR Egger
All − Inverse variance weighted
−2
−1
0
1
2
MR effect size for
'telomere length || id:ieu−b−4879' on 'IDP_dMRI_TBSS_MD_Posterior_limb_of_internal_cap
